# An Exploratory Study of Biceps Brachii Electromyographic Activity During Traditional Dumbbell Versus Bayesian Cable Curls

**DOI:** 10.3390/muscles4040045

**Published:** 2025-10-13

**Authors:** Koulla Parpa, Antreas Vasiliou, Marcos Michaelides, Karuppasamy Govindasamy, Anton Chernov, Konstantina Intziegianni

**Affiliations:** 1 School of Sciences, University of Central Lancashire—Cyprus Campus, University Avenue 12–14, 7080 Pyla, Cyprus; antreasvasiliou08@gmail.com (A.V.); mmichaelides@uclan.ac.uk (M.M.); ancher450259@gmail.com (A.C.); kintziegianni@uclan.ac.uk (K.I.); 2 Department of Sports Recreation and Wellness, Symbiosis International (Deemed University), Hyderabad Campus, Modallaguda, Nandigama, Rangereddy Dist., Hyderabad 509217, India; gowthamadnivog@gmail.com

**Keywords:** muscle activation, amplitude, electromyography, biceps brachii

## Abstract

Although previous studies have examined various factors that influence biceps brachii activation, such as grip position, load, and exercise variation, to our knowledge, no prior studies have compared muscle activation during a traditional biceps curl and a Bayesian cable curl. Therefore, this study aimed to examine the differences in biceps brachii muscle activation between these two training modalities. Data from eleven volunteers (age: 25 ± 6 y; weight: 86 ± 13 kg; height: 177 ± 8 cm) were included in the analysis. Muscle activity was assessed using the normalized root mean square (RMS) values obtained from surface electromyography (sEMG). A within-subjects, counterbalanced design was utilized where all participants completed both testing conditions in a randomized order to control for potential order effects. Participants visited the laboratory and fitness center on two occasions. On the first day, anthropometric measurements were obtained, along with one repetition maximum (1-RM) for both the dumbbell biceps curl and the Bayesian curl. On the second day, participants performed an isometric maximal voluntary contraction (MVC), followed by electromyographic assessment of muscle activity during the dumbbell biceps curl and the Bayesian curl, each performed at 80% of their respective 1-RM. When the normal distribution was confirmed via the Shapiro–Wilk test (*p* > 0.05), a paired *t*-test was used for statistical analysis. On the other hand, when normality was not confirmed, the Wilcoxon test was utilized. Statistically significant differences (*p* = 0.003) were observed in the EMG amplitude (%) between the biceps curl (111.46 ± 26.80) and the Bayesian curl (93.39 ± 15.65) with a large effect size (d = 0.82). Based on the EMG analysis, the dumbbell biceps curl elicited significantly greater muscle activation compared to the Bayesian curl, suggesting that the conventional movement places a higher mechanical and neuromuscular demand on the biceps brachii.

## 1. Introduction

Electromyography (EMG) research on biceps activation is essential for understanding how different resistance exercises influence muscle recruitment patterns [1]. Such insights support evidence-based exercise selection for optimizing strength and hypertrophy programs, as well as for rehabilitation and sports performance enhancement in both general and sports-specific contexts [2,3,4]. Among upper limb resistance exercises, the biceps curl is one of the most commonly used. It primarily involves elbow flexion accompanied by either dynamic or mostly isometric shoulder flexion and wrist supination or pronation [5]. As a result, the main muscles activated include the elbow flexors, shoulder flexors and wrist rotators.

Several studies have examined EMG activity of the elbow flexors using different exercise variations. Oliveira et al. (2009) [6] investigated the effect of shoulder position on muscle activation of the biceps brachii, using the EMG when executing different dumbbell curls (dumbbell biceps curl, incline dumbbell curl and dumbbell preacher curl). Their results suggested that when the arm is flexed isometrically at various angles, the greatest activation of biceps brachii occurs at a longer muscle length. At the same time, the dumbbell preacher curl showed high activation only at the beginning of the concentric phase with a significant decrease in EMG amplitude as the elbow flexed [6]. Concurrently, the investigators reported that during the eccentric phase, all exercises revealed reduced muscle activity, but the dumbbell biceps curl and incline dumbbell curl maintained a more consistent activation than the dumbbell preacher curl [6].

With regard to wrist positioning, Marcolin et al. (2018) [7] investigated the EMG activity of biceps brachii and brachioradialis during three common curl variations (dumbbell curls, straight barbell curls and EZ-bar curls). Their findings indicated that the EZ-bar curl elicited significantly higher biceps brachii and brachioradialis activations compared to the dumbbell curl throughout the entire movement. Also, during the concentric phase, brachioradialis activity was higher in EZ-bar curls and straight barbell curls compared to the dumbbell curls, and during the eccentric phase, both muscles had greater activation in EZ-bar curls than in dumbbell curls. The authors suggested that grip type and bar selection can influence muscle recruitment, with the EZ-bar being a more effective option for targeting biceps brachii and brachioradialis [7]. In a different study by Coratella and colleagues [8], it was indicated that biceps brachii activation was greater during the straight bar curl than the EZ bar curl.

Different curl variations were also investigated by Bagchi and Raizada (2019) [9], who compared the muscle activity of biceps brachii and brachioradialis during eight different types of biceps curl exercise. The investigators reported that concentrated curls produced the greatest activation of the biceps brachii, while hammer curls showed the greatest activation of the brachioradialis. At the same time, they demonstrated no difference between the biceps brachii curls and brachioradialis while performing the dumbbell curl with a supinated and neutral handgrip at different inter-hand distances. More recently, Coratella and colleagues (2023) [1] demonstrated that biceps brachii and brachioradialis activation were the highest with the supinated grip during the ascending phase of biceps curls, while the anterior deltoid showed greater activation with pronated and neutral grips. Concurrently, muscle activity was generally lower during the descending phase across all muscles and grip types.

In addition to the studies above, Signorile et al. (2017) [10] compared muscle activation and joint kinematics between cable-based and selectorized resistance machines during different exercises, including biceps curls. The investigators indicated that cable-based machines required greater activation of the stabilizing muscles, likely due to the increased demand for postural control and balance requirements imposed by the freedom of movement and gravitational challenges. In contrast, selectorized machines offered more stability and resulted in greater muscle activation of the primary movers.

While the aforementioned studies examined how variations in shoulder position, grip type and equipment selection influence EMG activation of the biceps brachii, brachioradialis and anterior deltoid muscles, no study has investigated the Bayesian cable curl. This exercise differs from traditional dumbbell curls because the cable’s posterior anchor point and hip-hinged body positioning alter the line of resistance, shoulder angle and length–tension relationship of the biceps. Unlike dumbbell curls, where resistance follows a vertical gravitational path, the Bayesian curl produced a backward and downward resistance vector, requiring the lifter to maintain shoulder extension while simultaneously resisting cable traction. These biomechanical differences may not only affect biceps activation but also change the coordination between the brachioradialis and the anterior deltoid. Considering the increasing popularity of Bayesian cable curls in strength training contexts, a comparison with the traditional dumbbell curl is important. Therefore, the purpose of this study is to examine differences in EMG activity between these two exercise variations. Based on prior findings that cable systems require greater stabilizer recruitment [10] and that muscle activation is influenced by shoulder position and grip type [6,7,8,9], we hypothesize that the Bayesian cable curl will produce lower activation of the primary movers compared to the traditional dumbbell curl, due to the increased postural demands of the exercise.

## 2. Materials and Methods

Participants

A total of thirteen participants with previous experience in weightlifting were recruited for the study. However, EMG recordings from two participants were excluded due to excessive signal noise, which was detected during data processing. Thus, the data from eleven volunteers (age: 25 ± 6 years; weight: 86 ± 13 kg; height: 177 ± 8 cm) were included in the analysis. Participants were recruited through advertisements placed in local fitness centers as well as through direct engagements with students enrolled in sports science courses. This constituted a convenience sample, as recruitment was based on accessibility and willingness to participate. To be eligible to participate in the study, individuals had to be healthy and physically active, as determined by their medical history, without any reported upper-body injuries, and within the age range of 20–40 years. Furthermore, only male participants who had engaged in weight training at least three times per week for a minimum of one year prior to the study were considered eligible. Participants who did not meet these inclusion criteria were excluded from the study. The study was conducted in accordance with the Declaration of Helsinki (World Medical Association, 2013) and was approved by the National Committee of Bioethics (CNBC, ΕΕΒΚ ΕΠ 2022.01.290). All participants provided written informed consent before data collection.

Study design

A within-subjects, counterbalanced design was utilized where all participants completed both testing conditions in a randomized order to control for potential order effects. Participants were asked to visit the laboratory and fitness center twice. On both testing days, which were scheduled one week apart, participants reported adherence to pre-assessment guidelines. Specifically, they confirmed maintaining adequate hydration, abstaining from alcohol the day before testing, and refraining from tobacco consumption on testing days, as well as avoiding any strenuous physical activity during the 24 h preceding each testing session. Furthermore, all participants confirmed that they had been habitually training with both dumbbells and cables and that they were right-hand dominant. On the first day, anthropometric measurements were obtained, along with one repetition maximum (1-RM) for both the dumbbell biceps curl and the Bayesian curl. On the second day, the participants performed an isometric maximal voluntary contraction (MVC) followed by the assessment of muscle activity using electromyography (EMG) during the dumbbell biceps curls and the Bayesian curl, both performed at 80% of their respective 1-RM.

Procedures and instrumentation
Anthropometric measurements and 1-RM

Stature and body mass were measured to the nearest 0.1 cm and 0.1 kg, respectively, using a wall-mounted stadiometer (The Leicester Height Measure, Tanita, Tokyo, Japan) and a calibrated electronic scale. The same investigator took these measurements with participants wearing minimal clothing and barefoot. Thereafter, the participants performed the 1-RM testing for both the dumbbell biceps curl and the Bayesian curl in a randomized order. The protocol recommended by the American College of Sports Medicine (ACSM) was utilized to determine the 1-RM for each exercise [11]. Participants first completed a light warm-up consisting of 5–10 repetitions at approximately 40–60% of their perceived maximum effort. After a one-minute rest period, they performed 3–5 repetitions at 60–80% of their perceived maximum. Thereafter, the load was progressively increased in small increments, and participants were asked to perform a single repetition in a proper form until concentric failure occurred. Adequate rest (3 min) was provided between attempts to ensure recovery and accuracy of the 1-RM determination. Upon completion of the 1-RM test for the first exercise, participants were given a standardized 3 min rest period before performing the 1-RM test for the second exercise, which was performed in a randomized order. Although previous researchers have suggested that the duration of the rest period does not significantly affect the accuracy of 1-RM determination, a standardized three-minute rest interval was implemented in the current study, in line with established recommendations [12]. This rest period was considered appropriate to ensure recovery between maximal efforts, minimize fatigue-related performance decrements and enhance consistency across participants and testing conditions. Two experienced investigators visually monitored trunk movements and knee flexion to ensure the proper execution of both exercises. For the appropriate execution of the biceps curl, participants performed the exercise while standing upright, with feet shoulder-width apart, holding a dumbbell in each hand in a supinated grip, as presented by previous investigators [6]. The arms were fully extended at the sides, with the elbows kept close to the torso throughout the whole movement. From this position, participants flexed their elbows to lift the dumbbells towards their shoulders in a controlled manner, avoiding trunk movements and jerky motions. The eccentric phase also involved a slow, controlled lowering of the dumbbells back to the starting position through the full range of motion. Regarding the execution of the Bayesian cable biceps curl, the participants had to stand facing away from the cable machine with the pulley set in a low position. This exercise has been previously described by investigators [10]. The dominant hand held the cable handle while the opposite arm was placed on the torso or hip for balance and support. The shoulder of the working arm was positioned in a slight extension, placing the biceps in a lengthened position at the beginning of the movement. From this position, the elbow was flexed to bring the hand toward the shoulder, followed by a controlled eccentric phase back to the starting position. Photos for both exercises are included in the appendices.

MVC and EMG testing

One week after the initial assessment, participants returned to the laboratory to perform an isometric maximal voluntary contraction (MVC), followed by the evaluation of muscle activity using surface electromyography (EMG) during the dumbbell biceps curls and the Bayesian curl, both performed at 80% of their respective 1-RM. To ensure consistency and reduce potential variability due to circadian influences on neuromuscular performance, all testing was conducted at the same time of day as the initial assessments.

All participants performed a standardized warm-up, similar to the one they followed during the initial testing. Thereafter, the skin over the biceps brachii belly on the participant’s dominant arm was shaved and disinfected with alcohol. An electromyography sensor (Delsys Inc., Boston, MA, USA, Trigno Avanti Sensor) was then positioned parallel to the orientation of the muscle fibers following the SENIAM recommendations [13]. All tests were carried out by the same investigator.

To determine the MVC of the elbow flexors, participants were seated on a stable bench with the shoulder in a neutral position and the elbow flexed at 45° on a fixed surface with the use of an electrical goniometer and their arm and forearm in a supinated position. A standard gym cable machine with adjustable weight stacks was used to provide resistance. The participant’s forearm was positioned parallel to the ground, holding the cable handle in a fixed position to ensure an isometric contraction. The pulley height was adjusted so that the resistance was applied directly along the line of elbow flexion. Delsys’s surface electromyography sensor was placed on the biceps brachii muscle belly longitudinally to muscle fibers, following the recommendations [13], and a goniometer (Guymon Goniometer, Model 01129, Lafayette, IN, USA) was used to confirm that the elbow joint remained at the correct position. Each participant performed two maximal isometric contractions, each held for 5 s, while the electrical activation of the biceps brachii was recorded via EMG works acquisition software (Delsys, v 4.8.0). A five-minute rest period was provided between the attempts to prevent fatigue. The highest recorded force was used to present the 100% MVC. The participants were instructed to pull the non-moving handle without lifting their elbow from the surface or changing its position, while verbal encouragement was provided to all of them.

Following the MVC testing, the participants rested for 5 min, according to the guidelines, to allow the muscle to completely recover [14]. Thereafter, they performed the biceps curl test and the Bayesian cable biceps curl in a randomized order following the same technique used during the initial testing at 80% of their 1-RM for both exercises. For each exercise, they had to perform six repetitions. For the Bayesian biceps curl, the pulley was set at hip height with a single D-handle, which provides the intended resistance profile for the biceps. The handle was rigid (with negligible compliance), and participants stood with their torso inclined forward at 10–150 degrees while keeping the working elbow positioned behind the torso to ensure consistent and reproducible execution. The range of motion for both exercises was verified during practice trials, and a qualified investigator supervised all sessions to ensure consistent execution across conditions. In addition, a metronome was set to 2 s for the concentric phase, 1 s for the isometric phase, and 3 s for the eccentric phase to control movement speed.

To assess muscle activation in the two conditions, the EMG signals were normalized to the values obtained during the maximum isometric contraction phase [15]. Data acquisition was performed using the Delsys EMG Trigno Wireless EMG system (Delsys Inc., Boston, MA, USA), connected to a PC running the Delsys EMGworks Acquisition software (version 4.8.0). The recorded signals were subsequently processed with Delsys EMGworks Analysis software (version 4.7.3.0) using a fourth-order Butterworth band-pass filter (20–450 Hz) and a sampling rate of 1259.26 Hz, which represents the system’s default setting.

The system also features a common mode rejection ratio > 80 dB and an input noise level < 0.75 µV RMS, providing a high signal-to-noise ratio and ensuring reliable EMG acquisition. Following data normalization, the root mean square (RMS, (mV)) computations were performed using a window length of 0.125 s with 0.0625 s of overlap. The specific window length corresponds to the default configuration of Delsys software. The primary outcome variable was EMG amplitude (%), as it reflects the magnitude of activation during a given activity relative to its maximal muscle activation [16,17,18].

Statistical analysis

All statistical analyses were performed using the SPSS software (SPSS Statistics, version 26, IBM, New York, USA). Results are reported as mean ± Standard deviation. Effect sizes were calculated using Cohen’s d for each *t*-test pairwise comparison [19]. Cohen’s d was interpreted as small (0.2–0.49), medium (0.5–0.79) and large (above 0.80). When normal distribution was confirmed via the Shapiro–Wilk test, a paired *t*-test was used for the statistical analysis. On the other hand, when normality was not confirmed, the Wilcoxon test was utilized. A post hoc analysis was performed utilizing G*power software (version 3.1.9.4) to compute the achieved power based on the sample size of the group. Based on the number of measurements and the calculated effect size (Cohen’s d = 0.82), the achieved power (1-β err prob) was 0.81. The level of statistical significance was set at α = 0.05.

## 3. Results

The anthropometric characteristics and 1-RM values of the eleven participants are presented in Table 1.

**Figure 1:**
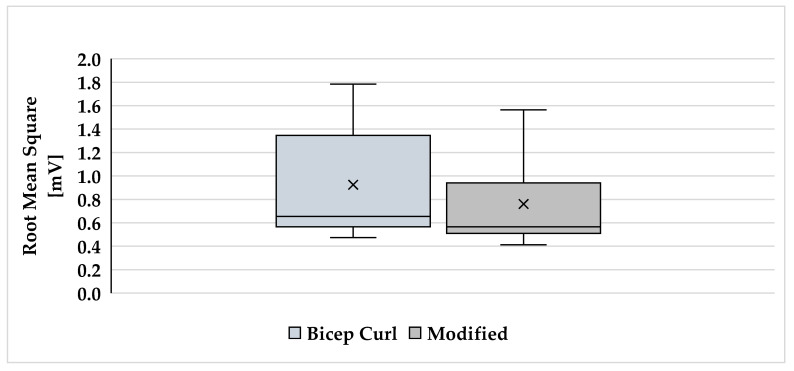
RMS of the two exercises; x = mean.

**Figure 2:**
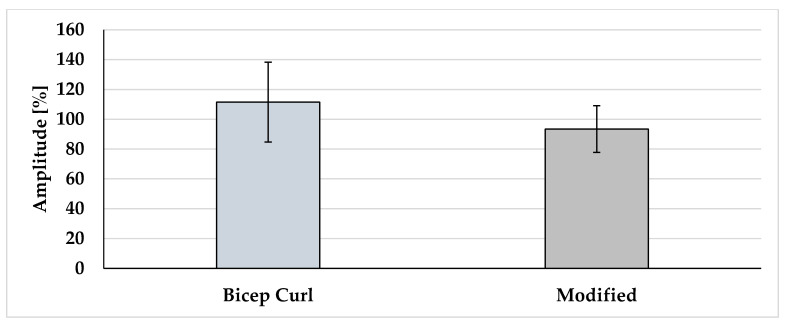
Amplitude of the two exercises.

The descriptive values (mean ± SD) for RMS and EMG amplitude (%) for each biceps exercise are graphically demonstrated in Figure 1 and Figure 2.

Statistically significant differences [t(10) = 3.96, *p* = 0.003] were observed in the EMG amplitude (%) between the biceps curl (111.46 ± 26.80) and the Bayesian curl (93.39 ± 15.65) with a large effect size (d = 0.82).

## 4. Discussion

This study compared neuromuscular activation of the biceps brachii during the traditional dumbbell biceps curl and the Bayesian cable curl. Considering the growing popularity of the Bayesian variation in strength training, the present findings offer practical insights into how these modalities influence muscle activation, with practical implications for exercise selection and program design. Based on the EMG analysis, the traditional dumbbell curl elicited significantly higher biceps brachii activation compared to the Bayesian curl, suggesting that the conventional exercise places greater mechanical and neuromuscular demand on the target muscle. These results are consistent with those of Signorile et al. (2017) [10], who reported that cable-based exercises require greater engagement of stabilizing muscles due to increased postural demands, resulting in lower activation of the primary movers.

When comparing the two exercise modalities, the traditional biceps curl requires moderate stability as the lifter stabilizes the torso while keeping the elbows by the sides. On the contrary, during the Bayesian curl, the backward pull from the cable increases postural challenge, especially on the shoulder girdle; therefore, a lower activation of the biceps brachii may occur. Concurrently, the free-weight curl, with its vertical resistance, provides a more stable base of support and facilitates greater control over the range of motion. On the other hand, the Bayesian curl involves a horizontal-diagonal resistance vector and requires greater control due to the starting position in shoulder extension. Although not directly comparable, our findings align with those of Coratella et al. (2023) [1], who reported higher biceps brachii activation with a supinated grip, suggesting that improved stabilization at the level of the humeral head facilitates greater biceps brachii activation. While both exercise variations in our study used a supinated grip, the enhanced stability during the traditional biceps curl likely contributed to the significantly higher activation of the biceps brachii.

The reduced activation during the Bayesian curl may also be partially explained by the muscle length–tension relationship [20]. In the starting position of the Bayesian curl, the long head of the biceps brachii is in a more lengthened state. According to the length–tension relationship, skeletal muscle generates maximal force at an optimal sarcomere length. Therefore, both excessive shortening and excessive lengthening can reduce the overall interaction between actin and myosin filaments, thereby decreasing force output [20]. Consequently, the elongated starting position of the Bayesian curl may reduce the muscle’s activity to generate high force, which is reflected in the lower EMG amplitude observed in this study. It should be noted that this study did not measure joint angles or directly quantify muscle length, so it remains a theoretical explanation.

Although initiating a movement with the biceps in a lengthened position, as in the Bayesian curl, has been suggested to enhance hypertrophic signaling [21], the lower EMG activation recorded here indicates that mechanical tension may not be effectively distributed to the biceps brachii in this exercise. Instead, tension may be shared with other muscles, reducing the neuromuscular demand on the primary mover. This highlights the importance of considering not only the type of exercise and the target muscles but also the body position in which the exercise is performed (standing, seated or lying down), as it can influence muscle activation and stabilization demands.

Unlike the standing variations in cable and free weight exercise discussed earlier, the study by Rosa and Colleagues [22] compared lying variations in free-weight and cable exercises [dumbbell bench press (DBP) vs. cable bench press (CBP)] and examined EMG activation of the pectoralis major, anterior deltoid, biceps brachii and triceps brachii. They indicated no significant differences in the pectoralis major and anterior deltoid activation between the two exercises, but the CBP resulted in greater activation of biceps brachii, whereas the DBP resulted in a greater activation of triceps brachii. Therefore, the difference in stabilization in this lying-down variation does not cause any differences in EMG activity in the primary movers, while it causes differences in the activation of secondary or stabilizing muscles. This suggests that while both modalities similarly engage the primary movers, the type of external resistance influences how supporting muscles are recruited to maintain movement stability and control.

## 5. Conclusions

We demonstrated greater EMG amplitude in the dumbbell biceps curl compared to the Bayesian biceps curl. This indicates that, in the context of acute neuromuscular activation, the traditional curl places greater demand on the biceps brachii. While higher activation is often interpreted as a potential indicator of greater hypertrophic stimulus, the present study only assessed acute responses and cannot confirm whether these differences translate into long-term adaptations in muscle size or strength. Therefore, longitudinal studies are needed to determine whether differences in activation translate into differences in strength or hypertrophy outcomes. From an applied perspective, these findings suggest that the traditional curl may be useful when the goal is to maximize immediate biceps activation, while the Bayesian curl may provide complementary benefits by introducing variation and increasing stabilization demands.

Furthermore, several limitations should be acknowledged when interpreting these findings. First, the sample size was small and consisted only of male participants, which limits the generalizability of the results to females and other age groups. Future studies should recruit larger and more diverse samples, and a priori power calculation is recommended to ensure adequate statistical power. Second, the analysis focused solely on the EMG activity of the biceps brachii, without accounting for other key elbow flexors such as the brachialis and brachioradialis. As these muscles contribute substantially to elbow flexion, future research should incorporate their activity to provide a more comprehensive understanding of recruitment patterns during both the traditional and Bayesian curls. Lastly, it is recommended that future studies incorporate kinematic analysis or 3D motion capture to determine the exact joint angles and muscle lengths at the start of each exercise, thereby clarifying their influence on EMG activation.

## Figures and Tables

**Table 1:** Anthropometric characteristics and 1-RM (Mean ± SD, n = 11).

Parameter	Mean ± SD	Minimum	Maximum
Age (years)	25.36 ± 6.31	20	40
Weight (kg)	86.91 ± 13.81	59	102
Height (cm)	177.36 ± 8.55	163	193
BMI (Kg.m^−2^)	27.44 ± 2.42	21.7	29.6
Dumbbell curl (kg)	22.73 ± 4.22	16	30
Bayesian curl (kg)	42.27 ± 12.92	25	65

Note: BMI: body mass index.

## Data Availability

Data are available upon request to the corresponding author.
